# The Application Prospects of Agarose as a Novel Skin Filler: A Narrative Review

**DOI:** 10.1111/jocd.70970

**Published:** 2026-06-09

**Authors:** Jianwen Gao, Yulong Xie, Xin Zhang, Yandong Liu, Peipei Xia, Zehua Wang

**Affiliations:** ^1^ College of Health Management, Shanghai Jian Qiao University Shanghai China; ^2^ Department of Health Science, Graduate School of Medicine Osaka University Osaka Japan; ^3^ BETAGENE Genetic INC. Shanghai China; ^4^ Petalregen INC. Shanghai China; ^5^ Obstetrics & Gynecology Hospital of Fudan University Shanghai Key Lab of Reproduction and Development, Shanghai Key Lab of Female Reproductive Endocrine Related Diseases Shanghai China

**Keywords:** agarose, degradation, dermal fillers, rheology

## Abstract

**Background:**

Agarose is a naturally derived polysaccharide that forms thermoreversible hydrogels with tunable rheological properties, making it a potential alternative platform to conventional dermal fillers. However, evidence regarding its physicochemical basis, preclinical performance, clinical outcomes, and the developmental status of commercially available agarose‐based filler products remains scattered across the laboratory and clinical literature. This narrative review aims to synthesize current progress in agarose‐based dermal fillers, spanning intrinsic material properties, experimental findings, and reported clinical product performance, to inform future research directions and product development.

**Methods:**

A literature search was performed in PubMed and Web of Science up to 31 February 2026. Keywords included agarose, hyaluronic acid (HA), calcium hydroxyapatite (CaHA), collagen, poly‐L‐lactic acid (PLLA), dermal fillers, rheology, biodegradation, and clinical outcomes. Eligible records included in vitro, ex vivo, in vivo, and clinical studies addressing agarose‐based filler formulation, structure–property relationships, degradation and clearance characteristics, biocompatibility, safety, and aesthetic effectiveness. Relevant comparative studies involving HA, CaHA, collagen, or PLLA were also included. Evidence was narratively summarized with emphasis on consistency, limitations, and translational relevance.

**Results:**

Available evidence indicates that agarose hydrogels provide tunable viscoelasticity and cohesive gel behavior through concentration‐dependent network formation, thereby supporting injectability and shape retention when appropriately formulated. Reported preclinical and limited clinical data suggest acceptable local tolerability. However, the clinical evidence base remains small and heterogeneous, with insufficient standardized correlations between rheological properties and clinical outcomes, limited head‐to‐head comparisons with established fillers, and inadequate long‐term data on biodegradation and rare delayed adverse events.

**Conclusions:**

Agarose represents a promising dermal filler platform supported by controllable rheological properties and generally favorable biocompatibility in existing studies. Future research should focus on standardized material characterization, controlled comparative clinical trials, and long‐term safety evaluation to better define its indications and comparative value.

**Trial Registration:**

Chinese Clinical Trial Registry ChiCTR2100046013. Sponsor: Lanzhou Biotechnique Development Co. Ltd (888 Yanchang Road, Chengguan District, Lanzhou, Gansu, China). Information provided by Zhou Jia (Applicant) and Prof. Luo Shengkang (Principal Investigator, The Second People's Hospital of Guangdong Province). Ethics approval: No. 2021‐QXLCYJ‐003‐03 (The Second People's Hospital of Guangdong Province Drug Clinical Trials Ethics Committee, approved 2021‐04‐08). Last Update Posted: 2021‐12‐13

## 1 Introduction

### 1.1 Background and Rationale

With the acceleration of global population aging and the increasing demand for aesthetic enhancements, dermal filler technology has gained a significant position in the field of aesthetic medicine. Dermal fillers are designed to correct wrinkles, depressions, and skin laxity, restoring skin firmness and youthfulness, and ultimately enhancing patients' appearance and self‐confidence. Common dermal filler materials include hyaluronic acid, collagen, and autologous fat. Although these materials have demonstrated specific clinical efficacy, they still have limitations, including a short duration of effect, allergic reactions, and immune rejection.

In recent years, with the rapid advancement of biomaterials technology, naturally derived polysaccharides have become a growing focus of research. Agarose, a natural polysaccharide extracted from red algae, has attracted increasing attention for dermal filling due to its excellent biocompatibility, tunable physical properties, and prolonged degradation profile [1]. Early preclinical and clinical reports suggest that agarose gels can provide volumization with generally acceptable short‐term tolerability [2]. However, comparative allergenicity and long‐term outcome data remain limited. Accordingly, agarose has been proposed as a candidate material for dermal filling, but its clinical positioning requires further standardized characterization and controlled comparative trials [3].

This review is timely because agarose‐based injectable gels have moved from material development toward early clinical use in selected markets. At the same time, the evidence remains fragmented across materials science, small clinical series, and regulatory summaries. A consolidated narrative synthesis can clarify what is supported by current data, what remains speculative, and where priority research is needed.

### 1.2 Review Methodology

This narrative review synthesizes heterogeneous evidence spanning agarose‐based injectable dermal filler formulation, rheological characterization, preclinical biocompatibility, and early clinical outcomes. Literature searches were conducted in PubMed, Web of Science and Google Scholar, supplemented by major Chinese‐language databases (e.g., CNKI, Wanfang Data, and NMPA) to capture both English‐ and Chinese‐language records (coverage to 28 February 2026). Search terms included “agarose”, “dermal filler”, “injectable”, “hydrogel”, “aesthetic”, “rheology”, “injectability”, “biocompatibility”, and “clinical outcomes”, along with comparator materials (“hyaluronic acid (HA)”, “calcium hydroxyapatite (CaHA)”, “collagen”, “poly‐L‐lactic acid (PLLA)”). Publicly available regulatory and product evaluation materials were additionally screened where accessible.

Eligible records included in vitro, in vivo and clinical studies reporting (i) Agarose‐based injectable gel formulations for soft‐tissue augmentation, (ii) Physicochemical properties relevant to clinical handling or tissue support, (iii) Clinical effectiveness or safety outcomes. Non‐relevant indications, non‐primary sources lacking analyzable data, and reports with insufficient methodological detail were excluded. Given substantial heterogeneity in formulations, testing conditions, indications, and endpoints, evidence was summarized qualitatively, organized by themes (material properties → rheology → biocompatibility → clinical applications), and interpreted with attention to comparators, follow‐up duration, outcome instruments, adverse‐event reporting, and limitations.

## 2 Fundamental Properties of Agarose

**FIGURE 1:**
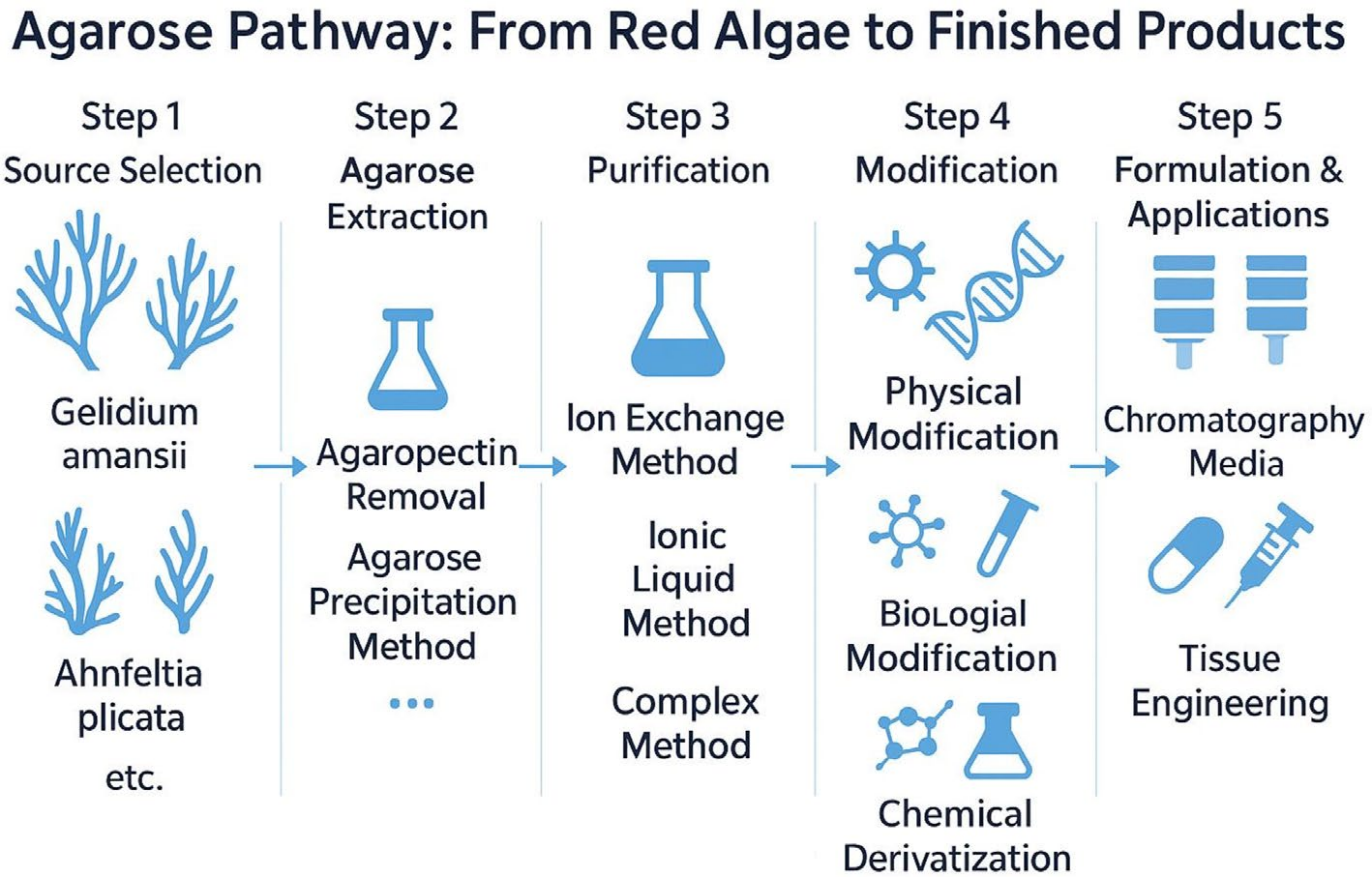
Agarose pathway: From red algae to finished products.

Agarose is a neutral natural polysaccharide extracted from red algae. Agar consists of agarose and agaropectin, from which agarose can be isolated through precipitation or ion‐exchange chromatography and subsequently purified (Figure 1). Due to its unique chemical structure and physicochemical properties, agarose has broad application potential in biomedicine, tissue engineering, and aesthetic medicine [4, 5, 6]. Agarose can function as a biofunctional carrier when combined with other materials, regulate swelling properties, and reinforce mechanical strength through its characteristic three‐dimensional network structure [7, 8, 9, 10].

### 2.1 Physicochemical Characteristics

**FIGURE 2:**
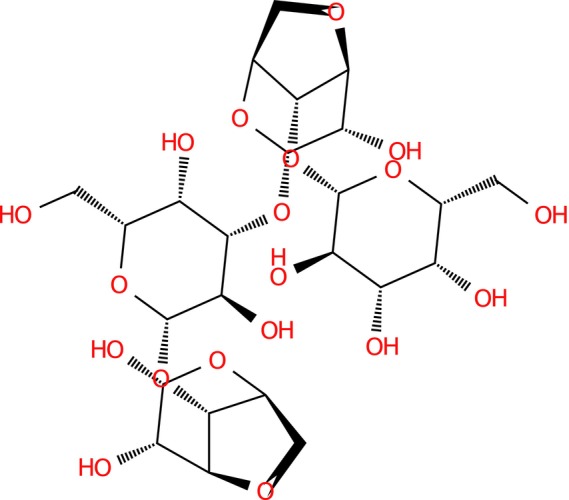
Agarose chemical structure.

Agarose comprises repeating units of 1,3‐β‐D‐galactopyranose and 1,4‐3,6‐anhydro‐α‐L‐galactopyranose linked alternately (Figure 2). This molecular architecture endows agarose with excellent hydrophilicity and gelling capability. The abundance of hydroxyl groups (–OH) along the polysaccharide backbone allows extensive hydrogen bonding with water molecules, thereby facilitating the formation of a stable hydrogel network in aqueous environments. This intrinsic property contributes to its high hydrophilicity and biocompatibility, representing a key feature that underpins its potential as a dermal filler material.

Agarose exhibits tunable physical characteristics under different conditions. The elasticity and viscosity of agarose hydrogels can be modulated by adjusting solution concentration and the degree of crosslinking [11, 12], thereby accommodating diverse filling requirements. Upon heating above 90°C, agarose dissolves in water; upon cooling to 35°C–45°C, it undergoes double‐helix self‐assembly to form a three‐dimensional hydrogel network, a process that exemplifies its thermoreversible gelation behavior [13, 14]. Gel strength is positively correlated with concentration: low concentrations (1%–3.5%) yield soft, transparent gels suitable for injectable fillers [15], whereas higher concentrations yield robust, stable gels suitable for implant‐type fillers. Moreover, chemical or physical crosslinking strategies can further enhance the stability and prolong the degradation profile of agarose hydrogels [16].

### 2.2 Rheological Properties

Agarose gels exhibit viscoelastic behavior relevant to injectability, in situ shape retention, and tissue support. Rheological parameters, particularly the storage modulus (G′) and loss modulus (G″), provide a quantitative description of elastic recovery versus viscous dissipation, which is frequently discussed in relation to the handling characteristics of injectable fillers [17, 18].

**FIGURE 3:**
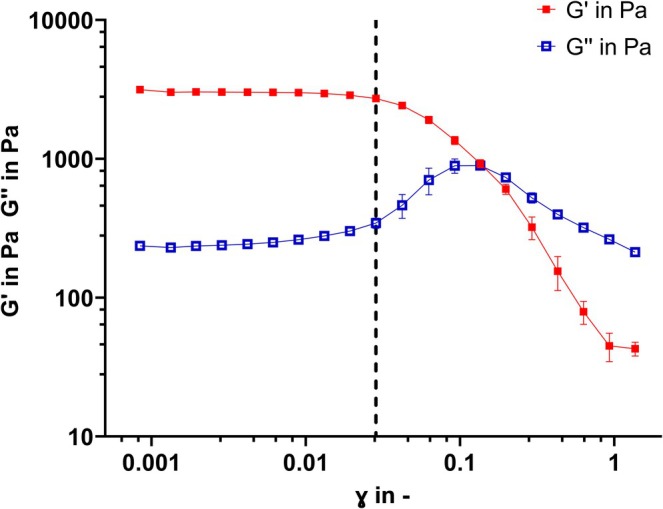
Oscillatory amplitude sweep of γ‐irradiated (25 kGy) 1.5% agarose gel filled in prefilled syringes, measured at 1 Hz and 25°C using a rotational rheometer with a 20‐mm parallel‐plate geometry and 0.5‐mm gap. Storage modulus (G′) and loss modulus (G″) are plotted as a function of strain across 20 *γ* points with triplicate measurements, illustrating a low‐strain viscoelastic plateau and a crossover at higher strain consistent with yielding behavior.

To provide agarose‐specific rheological evidence, a 1.5% agarose gel was filled into prefilled syringes and sterilized by γ‐irradiation at 25 kGy prior to testing. Rheological measurements were performed using a rotational rheometer (Thermo Scientific HAAKE MARS iQ Air) with a 20‐mm parallel‐plate geometry and a 0.5‐mm gap. The test was conducted at 25°C and 1 Hz, with the sample extruded using a 30G needle. The extrusion behavior of sample is shown in Video S1. An oscillatory amplitude sweep was collected across 20 strain (*γ*) points, with triplicate measurements per sample. As shown in Figure 3, the gel displayed a predominantly elastic response at low strain (G′ > G″) with a stable plateau. At *γ* ≈ 0.02845, G′ = 2719.6 ± 123.1 Pa and G″ = 344.7 ± 28.7 Pa (mean ± SD, *n* = 3). With increasing strain, G′ decreased markedly while G″ increased and then gradually declined; at *γ* ≈ 0.135, consistent with a transition toward a more dissipative, yielding‐like regime. These results document the strain‐dependent structural response of 1.5% agarose under the stated conditions. Due to differences in testing parameters and sample preparation procedures, agarose formulations of the same concentration may exhibit considerable variability in viscoelastic properties [19].

Agarose rheology is sensitive to preparation and processing, including thermal history, shear during mixing, and sterilization conditions; therefore, standardized reporting of test conditions and quality‐control–oriented rheological characterization are important for translational comparability across formulations [20, 21].

Beyond concentration adjustment, co‐crosslinking or blending agarose with other biomaterials has been reported to enhance mechanical support while introducing additional functionalities, including antibacterial activity, antioxidation, and wound‐healing potential [22, 23]. Finally, rheology should be interpreted in conjunction with formulation‐dependent attributes such as water uptake and gel cohesion, as materials with similar G′ and G″ values may still exhibit different handling characteristics and clinical behavior [17].

### 2.3 Recent Advances in Agarose Engineering

Agarose is chemically simple and therefore often requires functionalization to enable controlled crosslinking, bioactivity, and degradability. For polysaccharides lacking native carboxyl groups (including agarose), selective oxidation of primary alcohols to carboxylic acids introduces reactive handles for coupling, including peptide conjugation and incorporation of a crosslinkable motif. Click‐enabled approaches further provide modular post‐functionalization: installing azide or thiol groups enables CuAAC/SPAAC, thiol–ene, and related bioorthogonal reactions for rapid in situ functionalization and crosslinking [22]. Nanocomposite and double‐network (DN) architectures can mitigate the brittleness of neat agarose hydrogels while enabling composition‐dependent tuning of mechanical, swelling, and degradation properties [23].

#### 2.3.1 Co‐Crosslinking and Composite Design

Although agarose hydrogels are widely regarded as biocompatible, pure agarose systems can exhibit practical limitations that motivate co‐crosslinking and composite strategies, including variability in fibrous capsule thickness and neutrophil response, differences in capsule formation and degradation, and variations in bulk mechanical properties and material degradation rate [23]. Accordingly, incorporating secondary biomaterials (polymers, proteins, or inorganic phases) is frequently used to compensate for the shortcomings of neat agarose: bioactive composites are commonly described as having good biocompatibility and low immunogenicity, providing a supportive microenvironment for cells, reducing immune rejection, and promoting cell attachment, proliferation, and differentiation. Mechanistically, polymer–protein composites can improve biofunctionality by introducing cell‐adhesive motifs absent in agarose [24].

Composite selection also dictates degradation–mechanics coupling, which is central to functional performance. In agarose–ceramic systems, HA/agarose composites can provide high compressive strength suitable for load‐bearing contexts but may suffer from slow degradation that can impede remodeling, whereas β‐TCP/agarose scaffolds are described as rapidly degrading (reported 8–12 weeks) and promoting early osseointegration via ion release, albeit with reduced mechanical resilience and risk of premature structural collapse if degradation is too fast; therefore, optimizing HA/β‐TCP ratios is recommended to synchronize degradation with regeneration demands [23]. Rapidly formed HA or agarose gel composites have been explicitly noted as candidates for injectable biomaterials in orthopedic/oral/maxillofacial applications. At the same time, their slow degradation and limited inherent antimicrobial activity are discussed as potential constraints in dynamic or infection‐prone environments. Finally, crosslinking/co‐network strategies provide an additional lever for performance tuning in agarose composites. Crosslinked nanostructured fibrin–agarose hydrogels were reported to show biodegradability and improved biomechanical performance, with crosslinking regulating thickness, porosity, and fiber density to optimize both mechanical and biological performance, illustrating how composite architecture and crosslink density can be used to tune clinically relevant handling and function [25].

#### 2.3.2 Personalization Enabled by Formulation Tuning and Biofabrication

Agarose's adjustable physicochemical properties make it particularly suitable for customized therapies tailored to individual patient needs. By varying concentration and crosslinking degree, agarose can achieve different rheological properties to meet the requirements of various facial regions, ranging from fine‐line correction to deep contour volumization, thereby providing more precise and individualized treatment solutions.

“Personalized” agarose‐based treatments can be operationalized through CAD‐driven bioprinting and formulation‐level tuning to meet consumer requirements. In bioprinting, a key advantage is the ability to fabricate not only scaffolds with a specific, personalized shape, but also a designed internal network architecture supporting regeneration processes [26]. Agarose is frequently used as an injectable bioink due to its low processing temperature, rapid gelation, shear‐thinning behavior, and ability to support live cells. Practical customization can be achieved by adjusting agarose concentration to tune viscosity and printability, while recognizing that higher concentrations may reduce porosity and impair nutrient and waste transport. Moreover, carboxylated agarose (CA) enables finer material personalization: by regulating the degree of carboxylation, the elastic modulus of the printing gel can be precisely modulated (reported range 5–230 Pa) with minimal change in solution shear viscosity, allowing fabrication of 3D constructs with different mechanical domains under the same printing parameters and low nozzle shear stress [23]. Composite strategies also support personalization at the handling and performance level [26].

## 3 Application Scenarios and Development of Agarose‐Based Dermal Fillers

### 3.1 Facial Aesthetic Applications of Agarose

As a novel dermal filler, agarose demonstrates broad application potential in medical aesthetics and skin repair due to its excellent biocompatibility, tunability, and long‐lasting properties [27].

#### 3.1.1 Correction of Fine Lines and Wrinkles

Agarose injectables can be administered into facial fine lines or wrinkles to fill skin depressions. The hydrogel properties of agarose enable it to integrate seamlessly with dermal tissue, thereby providing a natural filling effect.

In a clinical study evaluating a nonhydrophilic agarose gel for facial rejuvenation, outcomes were assessed at 1 week using the Global Aesthetic Improvement Scale (GAIS) and a patient‐reported satisfaction survey (1–10 scale). In GAIS, 90% of cases were rated as “significant” or “great improvement,” and 83% of participants reported satisfaction ≥ 8/10; one late‐onset nodule was reported and resolved after corticosteroid injection [28]. Across reports, adverse reactions are typically localized and transient (e.g., erythema, edema, discomfort), although standardized, long‐term comparative data remain insufficient to define durability and safety profiles across indications [29].

#### 3.1.2 Facial Contouring and Volumization

Agarose injectables can also be used to augment facial depressions (e.g., cheeks, chin, temples) to restore three‐dimensional contours. Due to its softness and elasticity, agarose integrates well with skin tissue, providing natural volumizing effects.

In the published clinical experience (*n* = 700), follow‐up visits were scheduled at 1, 3, 6, 12, and 24 months; clinical improvement was evaluated using GAIS by two independent plastic surgeons at 1 year, and patient satisfaction was captured on a 0–10 scale. At 1 year, 82% of patients were rated as GAIS 1–2 (“exceptional” or “great improvement”), and 85% reported satisfaction ≥ 8/10 [15]. Given heterogeneity in injection technique, anatomical targets, and outcome reporting, controlled comparative trials with standardized endpoints are still needed to define evidence‐based indications and comparative value.

#### 3.1.3 Nonsurgical Rhinoplasty

Given the distinct characteristics of different filler materials, nonsurgical rhinoplasty procedures have employed combinations of hydrophilic hyaluronic acid and non‐hydrophilic, high G′ agarose gel as mixed injectables with complementary structural properties.

In one study, Agarose was injected supraperiosteally in volumes of 0.4–0.7 mL at the nasal radix and 0.4–0.6 mL along the nasal dorsum. Clinical outcomes were assessed 2 weeks post‐treatment using the Global Aesthetic Improvement Scale (GAIS; scored 1–5; 1 = exceptional improvement, 5 = worsened appearance). Patient satisfaction was evaluated using a 0–10 scale (0 = not satisfied, 10 = very satisfied). A total of 32 patients (mean age: 27 years) were enrolled. The mean satisfaction score was 9.09/10 immediately after injection and 9.0/10 at two weeks. GAIS scores were 1.72/5 immediately post‐injection and 1.69/5 at two weeks, indicating significant clinical improvement. No significant complications were observed during follow‐up [27].

#### 3.1.4 Skin Repair and Reconstruction

Agarose‐based hydrogels have also been investigated in wound‐related applications as space‐filling or supportive matrices that can maintain local architecture while tissue repair progresses. Because agarose degradation in vivo is relatively slow, it may provide prolonged structural support in some settings; however, most evidence for wound repair and scar modulation remains preclinical and does not yet justify definitive clinical claims.

Accordingly, statements regarding enhanced regeneration or reduced scarring should be interpreted as hypothesis‐generating. Mechanistically, agarose is not known to be degraded by a native human agarase; clearance is generally attributed to macrophage‐mediated processing and subsequent enzymatic pathways for oligosaccharide turnover, with additional agarase‐based degradation demonstrated in experimental contexts [30]. Well‐designed clinical studies with standardized endpoints are required before clinical effectiveness and safety in wound repair can be concluded.

### 3.2 Clinical Safety Evaluation

Safety evidence for agarose‐based dermal fillers is currently derived from observational clinical series, regulatory clinical trial datasets, and emerging preclinical models addressing high‐impact adverse outcomes. In real‐world clinical use, the largest available observational series (*n* = 700) reported infrequent complications, including infection (*n* = 1), palpable lumps (*n* = 5), and migration/displacement (*n* = 3) [15]. While no vascular occlusion or granuloma was reported in that cohort, rare events cannot be excluded, and the observational design, heterogeneous indications, and variability in injection technique and follow‐up constrain interpretation.

Regulatory clinical trial datasets provide more structured safety reporting under controlled conditions. A multicenter, randomized, parallel‐group, non‐inferiority trial (*n* = 258; maximum follow‐up 52 weeks) compared injectable agarose gel with a hyaluronic acid gel control for moderate‐to‐severe nasolabial folds. Overall, adverse‐event and serious adverse‐event rates were not significantly different between groups; however, injection‐site pain occurred more frequently in the agarose group (39.84% vs. 22.31%, *p* = 0.003) with a shorter mean duration (2.196 vs. 4.414 days) [28]. The technical review noted that the agarose arm used a larger‐gauge needle (27G) than the control arm (29G), which may contribute to the observed pain difference. By contrast, induration and erythema rates were not statistically different between groups (induration: 22.66% vs. 20.00%, *p* = 0.650; erythema: 7.81% vs. 8.46%, *p* = 1.000) [28]. These findings emphasize that technique‐ and device‐related factors (e.g., needle gauge) should be considered when interpreting tolerability signals and counseling patients.

Beyond human datasets, preclinical work has begun to examine embolic risk and dissolution characteristics relevant to severe filler complications. In a rabbit retinal artery embolism model, agarose gel was reported to reduce embolism risk compared with a hyaluronic acid filler under the experimental conditions, and dissolution testing using hyaluronidase suggested different dissolution behavior between materials [31]. These results are hypothesis‐generating and do not constitute an established clinical “reversal” strategy, but they provide a direction for standardized safety research focused on high‐impact adverse outcomes.

Overall, structured human safety data beyond 52 weeks remains limited. Longer‐term controlled comparative trials, harmonized adverse‐event definitions, and post‐market surveillance are needed to define the safety profile of agarose fillers across indications, injection planes, and procedural techniques.

### 3.3 Overview and Comparison of Dermal Fillers

To contextualize agarose‐based fillers within the broader field, it is important to review the current landscape of dermal filler materials and compare their physicochemical properties, clinical performance, and limitations (Table 1). The most commonly used fillers include hyaluronic acid, collagen‐based materials, autologous fat, poly‐L‐lactic acid (PLLA), and calcium hydroxylapatite (CaHA) [32, 33, 34, 35, 36].

**TABLE 1:** Comparative overview of injectable dermal filler materials.

Injectable filler material	Agarose	Hyaluronic acid (HA)	Collagen	Autologous fat	PLLA (poly‐L‐lactic acid)	CaHA (calcium hydroxylapatite)
Typical clinical duration	~6+ months (reported; varies with concentration/indication)	6 to 12 months, requires regular supplementation [37]	~3–6 months [38, 39]	Variable (depends on fat retention) [40]	~12–24 months [41, 42]	~12–18 months [43, 44]
Biocompatibility/safety	Reported as generally well tolerated; The occurrence of lumps or infections reported in clinical use showed no statistically significant difference compared to the control group [1].	Generally well tolerated; transient swelling/erythema/bruising; nodules possible [45, 46, 47, 48]	Allergy risk (often requires prior testing); immune reactions possible [49]	Biocompatible (autologous), but risks include infection, fat necrosis, cysts [50]	Biostimulatory; generally tolerated; nodules/granulomas possible if technique is suboptimal	Generally biocompatible; nodules possible; not enzyme‐reversible [51]
Adjustability	Moderate–High: tuning mainly via concentration and processing; formulation‐dependent handling.	High: wide product range; reversible with hyaluronidase	Low–Moderate (limited product tuning)	Moderate (volume can be adjusted; outcome less predictable)	Moderate (dose/session planning)	Moderate (immediate correction; fine‐tuning often needs additional treatment) [52]
Key limitations	Less clinic data; limited; technique sensitivity may affect palpability; long‐term (> 2 years) data still limited	Requires repeat treatments; risk of Tyndall effect in superficial planes; vascular occlusion risk (rare but severe) [53]	Short duration; allergy/immune risk; fewer modern indications compared with HA	Unpredictable survival/retention; may require multiple sessions; risk of serious vascular complications if injected improperly	Delayed visible effect; requires multiple visits and post‐treatment massage; complication management can be prolonged	Limited reversibility; not ideal for very superficial injections; complication management may be more difficult than HA

While these materials are widely applied in clinical practice, each exhibits characteristic trade‐offs related to durability, handling and injectability, reversibility, immunogenicity or allergic risk, and adverse‐event profiles. Against this backdrop, agarose has been explored as an additional hydrogel‐based option, and its properties and reported outcomes can be discussed alongside established fillers to delineate potential similarities and differences, as well as remaining evidence gaps [54, 55, 56].

#### 3.3.1 Comparison of Degradation Kinetics

Agarose gel, hyaluronic acid (HA) fillers, and poly‐L‐lactic acid (PLLA) exhibit distinct biodegradation kinetics in soft tissue.

Endogenous hyaluronidases primarily degrade HA fillers, whereas no native agarase has been identified in humans; accordingly, agarose clearance is thought to depend mainly on macrophage‐mediated phagocytosis, with subsequent lysosomal glycosidase activity (e.g., galactosidase) contributing to breakdown. In a longitudinal rat subcutaneous injection study assessed by MRI, agarose degradation/clearance was mainly attributed to macrophage‐mediated phagocytosis and mechanical fragmentation. In contrast, HA showed faster early volume loss, consistent with enzymatic degradation. Using the post‐injection month‐1 volume as 100%, agarose retained more volume than HA during the early phase (≤ 3 months: 86.24% vs. 61.88%, *p* = 0.004); HA volume reduction occurred chiefly within the first 3 months, while agarose showed a delayed decline mainly between 3 and 6 months, and residual volumes were not significantly different by 12 months. Agarose also demonstrated greater early dispersion than HA (≤ 3 months: 109.89% vs. 65.24%, *p* = 0.020), and neither material showed marked fibrosis or persistent inflammation at 12 months. In a clinical comparison, the mean duration was 257.5 ± 24.4 days for 2.5% agarose and 258.7 ± 24.5 days for NASHA‐L, with no statistically significant difference in persistence [11]. Separately, the clinical effectiveness of approximately 8 months for both 2.5% agarose gel and HA has been reported for correction of the nasolabial fold [57].

For clinical biodegradation metrics, two multicenter prospective cohorts of YVOIRE HA fillers defined “complete clinical biodegradation” by return to baseline wrinkle severity (WSRS change from baseline ≥ 0). They reported that HA effects typically persist 6–12 months, with complete clinical biodegradation reached in 93.5% and 98.5% of subjects within 104 weeks and in most subjects within 52 weeks after the last treatment [58].

In contrast, PLLA (PLA) is a bioresorbable aliphatic polyester with slower resorption; the randomized study reported a half‐life of ~31 days for L‐polylactides and an overall absorption time of ~18 months. PLA/PLLA was described as undergoing enzymatic and non‐enzymatic hydrolysis to lactic acid–related products that are metabolized (via the tricarboxylic acid cycle) and eliminated as CO_2_ and H_2_O, with a histologic sequence consistent with gradual resorption. Immune cell encapsulation at 3 months, porous deformation at 6 months, and minimal polymer residue by 9 months. Because PLLA is biostimulatory and promotes collagen neogenesis during resorption, its clinical aesthetic effect may persist for up to ~2 years [59].

## 4 Potential and Limitations

### 4.1 Potential

The global aesthetic medicine market is experiencing rapid growth, creating favorable conditions for the adoption and expansion of novel dermal filler materials such as agarose. As a naturally derived hydrogel platform, agarose has been explored as a dermal filler candidate; nevertheless, its evidence‐based positioning should be interpreted in light of limited comparative clinical data. To provide a clearer comparison of agarose‐based products with other representative mainstream filler products, Table 2 summarizes the company, region, composition, typical indications, regulatory/market status, and reversibility for each product.

**TABLE 2:** Overview of representative mainstream dermal filler products.

No.	Type	Representative products	Company	Region	Main composition	Typical indications	Regulatory/market status	Reversibility
1	Agarose‐based	Algeness family (VL/LD/HD/DF) [60]	Advanced Aesthetic Technologies Inc. (Manufactured by GHIMAS S.p.A.)	China + EU/Other	Concentration tiers: LD (agarose 1.0%); HD (agarose 1.5%); VL (agarose 2.5% + non‐crosslinked HA 0.5%); DF (agarose 3.5% + non‐crosslinked HA 0.4%)	LD: tear trough/lip augmentation; HD: wrinkles/lip contour; VL: mid‐to‐deep dermis/subcutaneous; DF: deep volume filling (chin/cheek, etc.)	China NMPA Class III Medical Device (Registration license No. 20253130037); EU: CE‐marked/marketed	No validated, established clinically methods for reversal
2	Agarose‐based	Agarose gel AG15	Betagene Genetic Inc.	China	Agarose 1.5%	Moderate‐to‐severe neck wrinkles	Pending certification/not yet filed	No validated, established clinically methods for reversal
3	HA	Juvéderm VOLUMA XC	Allergan Aesthetics	USA/global	Crosslinked HA gel (20 mg/mL) + lidocaine (0.3% w/w)	Deep injection for cheek augmentation/mid‐face volume deficit	US FDA approved (P110033)	Yes (HA fillers can be dissolved with hyaluronidase)
4	PLLA	Sculptra Aesthetic	Galderma	USA/global	Injectable PLLA particles + excipients (carboxymethylcellulose, mannitol, etc.)	Facial wrinkles/folds correction	US FDA approved (P030050)	Not be reversed without physical removal
5	CaHA	Radiesse	Merz Aesthetics	USA/global	Synthetic calcium hydroxylapatite suspended in gel carrier	Moderate‐to‐severe facial wrinkles/folds; also hand augmentation	US FDA approved (P050037)	Not be reversed without physical removal
6	PMMA	Bellafill	Suneva Medical	USA	20% PMMA microspheres +80% bovine collagen solution	Nasolabial folds; atrophic, distensible acne scars on the cheek	US FDA PMA (P020012)	Not be reversed without physical removal

#### 4.1.1 Rapid Growth of the Global Beauty Market

The global aesthetic market continues to expand steadily. According to recent statistics, the total number of aesthetic procedures is projected to reach nearly 38 million in 2024, representing an approximately 40% increase from 2020 [61]. Against this backdrop, agarose‐based fillers may represent an emerging option; market adoption will depend on robust clinical evidence, standardized quality metrics, and regulatory evaluation.

As the world's second‐largest economy, China's medical aesthetics market surpassed 300 billion yuan in 2020 and continues to expand rapidly [62]. With growing consumer demand for cosmetic treatments, particularly in first‐ and second‐tier cities, the dermal filler sector has demonstrated robust growth momentum. Agarose, owing to its natural composition and reduced risk of allergic reactions, has gained traction among domestic consumers and shows potential to occupy a unique niche in this growing market.

#### 4.1.2 Increasing Consumer Demand for Safety and Biocompatibility

With increasing awareness of filler‐related complications, safety and biocompatibility have become crucial factors in consumer decision‐making. Agarose, a naturally derived polysaccharide, exhibits low immunogenicity and non‐toxicity, making it an ideal alternative for patients who experience allergic reactions to traditional fillers such as hyaluronic acid or collagen [29].

The degradation rate of agarose directly influences its duration of effect and tissue stability. Although agarose exhibits slow, controllable degradation, optimization is necessary to meet diverse clinical needs [22]. Additionally, agarose can be selectively degraded by agarases extracted from various microbial strains, providing a potential means for controlled biodegradation and clearance in biomedical and cosmetic applications [63, 64, 65, 66]. This degradability feature enhances its clinical controllability and expands its potential for long‐term use in aesthetic medicine [67].

### 4.2 Limitations

Continuous advancements in biomaterial science and polymer engineering are driving innovation in agarose fillers. Through molecular design and crosslink optimization, researchers can further refine the degradation kinetics and mechanical behavior of agarose gels, thereby improving their injectability, tissue integration, and durability. These developments pave the way for next‐generation agarose‐based fillers that combine clinical efficacy with enhanced user experience.

Despite its remarkable potential, several technical and regulatory challenges must still be addressed before agarose‐based fillers can achieve broader clinical and commercial application.

#### 4.2.1 Technical Challenges

##### 4.2.1.1 Sterilization and Endotoxin Control

Sterilization of polysaccharide‐based hydrogels can be technically challenging because commonly used methods may alter polymer structure and performance. Gamma irradiation and ethylene oxide can induce unwanted crosslinking or depolymerization, whereas steam sterilization can promote hydrolytic chain scission; consequently, sterilization must be selected on a case‐by‐case basis to preserve material integrity and injectability. Importantly, while these approaches can inactivate bacteria and viruses, they do not remove endotoxin, which may provoke strong host responses; endotoxin detection and dedicated removal workflows are therefore critical quality controls for polysaccharide biomaterials intended for implantation or injection [22].

Sterilization and processing must be incorporated into this rheology‐to‐clinic translation, as they can measurably alter viscoelasticity even at “typical” sterilization doses. Electron‐beam irradiation of agarose hydrogels across 0–30 kGy results in a significant decrease in both storage and loss moduli; at 30 kGy, the viscoelastic properties decrease by > 20% relative to the initial moduli, attributed to partial network destruction and radiolytic degradation [68]. Therefore, for agarose fillers, it is defensible to propose a QC‐oriented workflow in which concentration is treated as the primary formulation lever. In contrast, the sterilization dose/method is treated as a critical secondary variable that must be controlled and verified by rheology before release [68]. Finally, the framework should remain explicitly “updatable”: composite or co‐crosslinked agarose systems can preserve or reshape mechanical profiles and structure–function relationships, and thus may change clinical behavior and degradation kinetics in ways that require dedicated future validation [24].

##### 4.2.1.2 Injection Technique Variability

Clinical performance and complication profiles of agarose gel fillers depend not only on material properties but also on injection technique. In a large clinical experience, agarose gel was delivered using either a blunt‐tip cannula or needle, with technique, volumes, and concentrations varying across indications; injections were performed at different tissue planes, and both linear threading and bolus techniques were used. Given agarose gel's low migration tendency, linear threading has been recommended in practice to reduce lump formation, and immediate gentle massage/molding after injection is commonly used to optimize contour and correct early irregularities. Consistent with technique sensitivity, palpable but typically self‐resolving lumps have been reported during follow‐up, underscoring the need for standardized training, anatomical plane selection, and consistent post‐injection molding protocols [15].

##### 4.2.1.3 Potential for Biofilm Formation and Mitigation Strategies

Biofilm‐associated infection is a recognized mechanism underlying delayed or recurrent inflammatory complications after dermal filler procedures. It has been discussed extensively for hyaluronic acid fillers, where biofilm is described as a surface‐associated microbial community embedded in a polymeric matrix that increases tolerance to host defenses and antibiotics [69, 70]. Recent clinical‐pathology evidence also supports biofilm formation as a risk factor for late and delayed complications after filler injection, consistent with a contamination‐ and host‐triggered process rather than an immediate acute infection alone [71, 72].

For agarose fillers specifically, direct clinical evidence demonstrating biofilm formation on agarose implants/fillers is limited in the open literature; therefore, biofilm risk should be framed as a plausible, but not yet agarose‐specific, clinically established complication pathway. Mechanistically, however, agarose hydrogels are widely used as defined scaffolds in biofilm research, indicating that agarose can serve as a physical three‐dimensional matrix for bacterial organization when microorganisms are introduced [73]. Accordingly, in the context of dermal fillers, the most defensible interpretation is that biofilm risk for agarose is likely driven by procedural contamination, similarly to other filler materials, rather than by a material‐unique “biofilm‐generating” property [69].

#### 4.2.2 Production Cost and Industrial Scalability

The production process of agarose is relatively complex and costly, which limits its large‐scale market penetration. Compared with traditional filling materials, its higher unit cost may affect promotion in price‐sensitive markets. Additionally, large‐scale extraction and purification require advanced industrial equipment and rigorous quality control.

Future research should focus on improving production efficiency, reducing purification costs, and optimizing extraction technologies to enhance scalability and economic feasibility for commercial applications.

#### 4.2.3 Clinical Validation and Market Acceptance

Although current studies confirm the safety and efficacy of agarose‐based fillers, more long‐term clinical trials and post‐market surveillance are needed to provide robust evidence supporting their clinical value.

Moreover, market education and professional training are crucial to improve acceptance among physicians and consumers. Building confidence in agarose's safety profile and unique advantages compared with traditional fillers will be critical for its widespread adoption.

#### 4.2.4 Policy, Regulation, and Standardization

As an emerging biomaterial in aesthetic medicine, agarose‐based dermal fillers must comply with region‐specific regulatory frameworks, including those of China's National Medical Products Administration (NMPA), which emphasize safety evaluation, product traceability, and risk control. These requirements strengthen assurances of quality, safety, and clinical performance, but may also prolong development timelines and increase compliance costs.

At present, regulatory pathways for agarose fillers remain heterogeneous across jurisdictions; while clinical evidence supporting regulatory review is beginning to accumulate (e.g., multicenter clinical data reported in NMPA technical review documentation), broader regulatory positioning comparable to established filler categories, such as hyaluronic acid, is still evolving [74].

In this context, the development of standardized testing protocols, harmonized quality criteria, and clearer global certification routes, together with robust post‐market surveillance, will be important to support consistent evaluation and responsible clinical translation.

## 5 Summary and Outlook

Despite the wide availability of established dermal fillers such as hyaluronic acid, collagen‐based materials, calcium hydroxyapatite, poly‐L‐lactic acid, and autologous fat grafting, each class has practical constraints, including variable longevity, product‐ and technique‐dependent performance, and distinct safety profiles. These considerations have driven interest in alternative hydrogel platforms that may offer predictable mechanics, injectability, and durable yet biocompatible tissue responses. As a naturally derived polysaccharide capable of forming thermoreversible physical gels, agarose has emerged as a candidate dermal filler material whose performance can, in principle, be tuned through formulation and processing.

This narrative review integrated evidence spanning agarose's intrinsic material properties, laboratory and preclinical studies, and reported clinical performance of agarose‐based dermal filler products. Overall, the literature suggests that agarose hydrogels can provide concentration‐ and formulation‐dependent viscoelasticity and cohesive gel behavior through physically crosslinked network formation, supporting handling and shape retention when appropriately engineered. Preclinical and limited clinical reports generally describe acceptable local tolerability dominated by transient injection‐site reactions; however, the clinical evidence base remains relatively small and heterogeneous, with limited standardized endpoints, insufficient rheology–outcome mapping, and scarce head‐to‐head comparisons with established fillers.

Looking forward, several priorities are critical for strengthening translation and clinical adoption: (i) standardized characterization of critical quality attributes (e.g., oscillatory rheology, cohesivity, extrusion force, injectability, swelling, and structural recovery after shear) with transparent reporting to enable cross‐study comparability; (ii) mechanistic and quantitative studies of in vivo persistence and clearance, including how formulation and injection depth influence residence time; (iii) rigorous evaluation of safety risks relevant to injectable gels, including delayed nodules, inflammatory responses, and infection/biofilm considerations, as well as the Impact of sterilization and storage on gel structure and performance; and (iv) well‐designed comparative clinical trials with harmonized outcome measures and longer follow‐up, supplemented by post‐marketing surveillance or registry‐based monitoring where applicable.

In summary, agarose‐based fillers are a promising but still incompletely validated alternative within the dermal filler landscape. Mechanistic plausibility and early reports support concentration‐ and processing‐dependent tunability with generally acceptable short‐term local tolerability; however, the clinical evidence remains limited and heterogeneous, with scarce head‐to‐head comparisons and incomplete long‐term surveillance. Accordingly, the future clinical role of agarose will depend on standardized characterization linked to handling and outcomes, rigorous comparative clinical trials with harmonized endpoints, and long‐term safety and clearance monitoring. Until such data becomes available, agarose should be considered an emerging skin material option, and its ultimate value must be determined through stronger comparative clinical evidence.

## Author Contributions

Conceptualization, J.G. and Z.W.; methodology, Y.X. and Z.W.; software, X.Z.; validation, Y.X., Y.L. and P.X.; formal analysis, X.Z.; investigation, Y.X., Y.L. and P.X.; resources, J.G.; data curation, X.Z.; writing – original draft, J.G. and Y.X.; writing – review and editing, Z.W., J.G. and Y.X.; visualization, X.Z.; supervision, Z.W.; project administration, J.G.

## Funding

The authors have nothing to report.

## Ethics Statement

The authors have nothing to report.

## Consent

The authors have nothing to report.

## Conflicts of Interest

The authors declare no conflicts of interest.

## Supporting information


**Video S1:** Extrusion behavior of 1.5% agarose gel through a 30G needle. A prefilled syringe containing 1.5% agarose gel sterilized by γ‐irradiation at 25 kGy was fitted with a 30G needle and manually extruded to demonstrate the injectability and continuous delivery behavior of the gel.

## Data Availability

Data sharing not applicable to this article as no datasets were generated or analysed during the current study.

## References

[1] S. Fernandez‐Cossio , A. León‐Mateos , F. G. Sampedro , and M. T. C. Oreja , “Biocompatibility of Agarose Gel as a Dermal Filler: Histologic Evaluation of Subcutaneous Implants,” Plastic and Reconstructive Surgery 120, no. 5 (2007): 1161–1169. 17898590 10.1097/01.prs.0000279475.99934.71

[2] A. Scarano , F. Carinci , and A. Piattelli , “Lip Augmentation With a New Filler (Agarose Gel): A 3‐Year Follow‐Up Study,” Oral Surgery, Oral Medicine, Oral Pathology, Oral Radiology, and Endodontics 108, no. 2 (2009): e11–e15. 10.1016/j.tripleo.2009.04.025 19615639

[3] F. Campos , A. B. Bonhome‐Espinosa , J. Chato‐Astrain , et al., “Evaluation of Fibrin‐Agarose Tissue‐Like Hydrogels Biocompatibility for Tissue Engineering Applications,” Frontiers in Bioengineering and Biotechnology 8 (2020): 596. 32612984 10.3389/fbioe.2020.00596 PMC7308535

[4] F. Jiang , X. W. Xu , F. Q. Chen , et al., “Extraction, Modification and Biomedical Application of Agarose Hydrogels: A Review,” Marine Drugs 21, no. 5 (2023): 299. 37233493 10.3390/md21050299 PMC10220669

[5] O. Ortiz‐Arrabal , A. Irastorza‐Lorenzo , F. Campos , et al., “Fibrin and Marine‐Derived Agaroses for the Generation of Human Bioartificial Tissues: An Ex Vivo and In Vivo Study,” Marine Drugs 21, no. 3 (2023): 187. 36976236 10.3390/md21030187 PMC10058299

[6] M. Tabata , T. Shimoda , K. Sugihara , D. Ogomi , H. Ohgushi , and M. Akashi , “Apatite Formed On/In Agarose Gel as a Bone‐Grafting Material in the Treatment of Periodontal Infrabony Defect,” Journal of Biomedical Materials Research. Part B, Applied Biomaterials 75, no. 2 (2005): 378–386. 16034996 10.1002/jbm.b.30316

[7] G. K. Mamytbekov , I. V. Danko , Z. I. Beksultanov , Y. R. Nurtazin , and V. I. Bannych , “Synthesis and Characterization of Shungite Modified Poly‐N‐Ninylpyrrolidone‐Agarose Composites for Medical Application,” ACS Omega 9, no. 41 (2024): 42297–42308. 39431079 10.1021/acsomega.4c04828 PMC11483400

[8] E. Kolanthai , K. Ganesan , M. Epple , and S. N. Kalkura , “Synthesis of Nanosized Hydroxyapatite/Agarose Powders for Bone Filler and Drug Delivery Application,” Materials Today Communications 8 (2016): 31–40.

[9] J. Watanabe , M. Kashii , M. Hirao , et al., “Quick‐Forming Hydroxyapatite/Agarose Gel Composites Induce Bone Regeneration,” Journal of Biomedical Materials Research. Part A 83, no. 3 (2007): 845–852. 17559128 10.1002/jbm.a.31435

[10] A. D. Cigan , B. L. Roach , R. J. Nims , et al., “High Seeding Density of Human Chondrocytes in Agarose Produces Tissue‐Engineered Cartilage Approaching Native Mechanical and Biochemical Properties,” Journal of Biomechanics 49, no. 9 (2016): 1909–1917. 27198889 10.1016/j.jbiomech.2016.04.039 PMC4920373

[11] N. Scuderi , B. Fanelli , P. Fino , and B. M. Kinney , “Comparison of 2.5% Agarose Gel vs Hyaluronic Acid Filler, for the Correction of Moderate to Severe Nasolabial Folds,” Journal of Cosmetic Dermatology 20, no. 5 (2021): 1512–1519. 33533155 10.1111/jocd.13962 PMC8248355

[12] A. Ed‐Daoui , M. H. Benelmostafa , and M. Dahmani , “Elasticity and Conformational Structure of Pure and Modified Agaroses Gel,” Polymer Bulletin 79 (2022): 11119–11137.

[13] N. Jin , E. A. Morin , D. M. Henn , et al., “Agarose Hydrogels Embedded With pH‐Responsive Diblock Copolymer Micelles for Triggered Release of Substances,” Biomacromolecules 14, no. 8 (2013): 2713–2723. 23815070 10.1021/bm4005639 PMC3773184

[14] V. Crespo‐Cuevas , V. L. Ferguson , and F. Vernerey , “Poroviscoelasto‐Plasticity of Agarose‐Based Hydrogels,” Soft Matter 19, no. 4 (2023): 790–806. 36625244 10.1039/d2sm01356h PMC12050128

[15] O. Buhsem and A. Kirazoglu , “Agarose Gel: An Overview of the Dermal Filler and a Clinical Experience With 700 Patients,” Aesthetic Surgery Journal Open Forum 5 (2023): ojad051. 37700788 10.1093/asjof/ojad051 PMC10494782

[16] A. Awadhiya , D. Kumar , and V. Verma , “Crosslinking of Agarose Bioplastic Using Citric Acid,” Carbohydrate Polymers 151 (2016): 60–67. 27474543 10.1016/j.carbpol.2016.05.040

[17] C. de la Guardia , A. Virno , M. Musumeci , A. Bernardin , and M. B. Silberberg , “Rheologic and Physicochemical Characteristics of Hyaluronic Acid Fillers: Overview and Relationship to Product Performance,” Facial Plastic Surgery 38, no. 2 (2022): 116–123. 35114708 10.1055/s-0041-1741560 PMC9188840

[18] S. P. Fundaro , G. Salti , D. M. H. Malgapo , and S. Innocenti , “The Rheology and Physicochemical Characteristics of Hyaluronic Acid Fillers: Their Clinical Implications,” International Journal of Molecular Sciences 23, no. 18 (2022): 10518. 36142430 10.3390/ijms231810518 PMC9503994

[19] M. Ghebremedhin , S. Seiffert , and T. A. Vilgis , “Physics of Agarose Fluid Gels: Rheological Properties and Microstructure,” Current Research in Food Science 4 (2021): 436–448. 34258588 10.1016/j.crfs.2021.06.003 PMC8255179

[20] M. Babaluei , Y. Mojarab , F. Mottaghitalab , and M. Farokhi , “Injectable Hydrogel Based on Silk Fibroin/Carboxymethyl Cellulose/Agarose Containing Polydopamine Functionalized Graphene Oxide With Conductivity, Hemostasis, Antibacterial, and Anti‐Oxidant Properties for Full‐Thickness Burn Healing,” International Journal of Biological Macromolecules 249 (2023): 126051. 37517755 10.1016/j.ijbiomac.2023.126051

[21] Q. Wang , H. Yan , Y. Guo , B. Tian , and J. Xiao , “High‐Temperature Emulsification Coupled With Low‐Temperature Gelation for Fabrication of Agarose Microsphere Implants With Well‐Controlled Size for Skin Tissue Enhancement,” Journal of Materials Chemistry B 12, no. 42 (2024): 10983–10993. 39350564 10.1039/d4tb01564a

[22] M. Beaumont , R. Tran , G. Vera , et al., “Hydrogel‐Forming Algae Polysaccharides: From Seaweed to Biomedical Applications,” Biomacromolecules 22, no. 3 (2021): 1027–1052. 33577286 10.1021/acs.biomac.0c01406 PMC7944484

[23] Y. B. Huang , Y. Huang , S. Peng , Y. Chen , and B. Chu , “Agarose Hydrogels for Bone Tissue Engineering, From Injectables to Bioprinting,” Gels 11, no. 4 (2025): 23. 40277691 10.3390/gels11040255 PMC12027395

[24] C. Zigan , C. Benito Alston , A. Chatterjee , L. Solorio , and D. D. Chan , “Characterization of Composite Agarose‐Collagen Hydrogels for Chondrocyte Culture,” Annals of Biomedical Engineering 53, no. 1 (2025): 120–132. 39277549 10.1007/s10439-024-03613-x PMC11782374

[25] S. Karunanithi and K. Rajappan , “Biofunctionalized Agarose‐Based Biopolymer Composites for Advanced Biomedical Applications: A Review,” Polymer Bulletin 82, no. 10 (2025): 4835–4877.

[26] S. Mania , A. Banach‐Kopeć , N. Maciejewska , et al., “From Bioink to Tissue: Exploring Chitosan‐Agarose Composite in the Context of Printability and Cellular Behaviour,” Molecules 29, no. 19 (2024): 19. 10.3390/molecules29194648 PMC11477700 39407579

[27] O. Buhsem and A. Kirazoglu , “Hybrid Nasal Filler: Combining Agarose Gel and Hyaluronic Acid for Nonsurgical Rhinoplasty,” Plastic and Reconstructive Surgery. Global Open 10, no. 4 (2022): e4236. 35402124 10.1097/GOX.0000000000004236 PMC8987220

[28] Technical Review Report for Injectable Agarose Gel Facial Filler (Acceptance No. JQZ2300210) (National Medical Products Administration, 2025).

[29] C. Karapantzou , M. Jakob , B. Kinney , J. Vandeputte , J. P. Vale , and M. Canis , “The Use of Algeness in the Face and Neck: A Safe, Alternative Filler for Cosmetics and Reconstruction,” Ann Transl Med 8, no. 6 (2020): 362. 32355806 10.21037/atm.2020.02.52 PMC7186688

[30] C. Jiang , Z. Liu , D. Cheng , and X. Mao , “Agarose Degradation for Utilization: Enzymes, Pathways, Metabolic Engineering Methods and Products,” Biotechnology Advances 45 (2020): 107641. 33035614 10.1016/j.biotechadv.2020.107641

[31] Z. Liu , Y. Jiang , J. Zhou , C. Pan , and K. Liu , “Agarose Gel as an Injectable Filler Significantly Reduced the Risk of Rabbit Retinal Artery Embolism Compared to Hyaluronic Acid,” Aesthetic Plastic Surgery 50, no. 5 (2026): 2047–2053. 41634454 10.1007/s00266-026-05670-0

[32] B. L. Eppley and B. Dadvand , “Injectable Soft‐Tissue Fillers: Clinical Overview,” Plastic and Reconstructive Surgery 118, no. 4 (2006): 98e–106e. 10.1097/01.prs.0000232436.91409.30 16980841

[33] N. Zerbinati and A. Calligaro , “Calcium Hydroxylapatite Treatment of Human Skin: Evidence of Collagen Turnover Through Picrosirius Red Staining and Circularly Polarized Microscopy,” Clinical, Cosmetic and Investigational Dermatology 11 (2018): 29–35. 29391818 10.2147/CCID.S143015 PMC5772396

[34] S. Oh , S. B. Seo , G. Kim , et al., “Poly‐D,L‐Lactic Acid Filler Increases Extracellular Matrix by Modulating Macrophages and Adipose‐Derived Stem Cells in Aged Animal Skin,” Antioxidants (Basel) 12, no. 6 (2023): 1204. 37371934 10.3390/antiox12061204 PMC10294940

[35] T. Iannitti , J. C. Morales‐Medina , A. Coacci , and B. Palmieri , “Experimental and Clinical Efficacy of Two Hyaluronic Acid‐Based Compounds of Different Cross‐Linkage and Composition in the Rejuvenation of the Skin,” Pharmaceutical Research 33, no. 12 (2016): 2879–2890. 24962508 10.1007/s11095-014-1354-y

[36] G. Casabona , K. Kaye , S. Cotofana , K. Davidovic , M. Alfertshofer , and L. Freytag , “Histological Effects of a Combined Collagen Stimulation Procedure Consisting of Microfocused Ultrasound, Soft Tissue Filler, and ca‐HA Injections,” Journal of Cosmetic Dermatology 22, no. 6 (2023): 1724–1730. 37073423 10.1111/jocd.15770

[37] K. L. Beasley , M. A. Weiss , and R. A. Weiss , “Hyaluronic Acid Fillers: A Comprehensive Review,” Facial Plastic Surgery 25, no. 2 (2009): 86–94. 19415575 10.1055/s-0029-1220647

[38] A. P. Sclafani , T. Romo, III , A. Parker , S. A. McCormick , R. Cocker , and A. Jacono , “Homologous Collagen Dispersion (Dermalogen) as a Dermal Filler: Persistence and Histology Compared With Bovine Collagen,” Annals of Plastic Surgery 49, no. 2 (2002): 181–188. 12187346 10.1097/00000637-200208000-00011

[39] M. J. Rapaport , R. Salit , and L. Rivkin , “Collagen Injections for Aging Skin Lines (Wrinkles),” Journal of the American Academy of Dermatology 11, no. 2 Pt 1 (1984): 250–252. 6480926 10.1016/s0190-9622(84)70159-9

[40] L. P. Bucky and S. K. Kanchwala , “The Role of Autologous Fat and Alternative Fillers in the Aging Face,” Plastic and Reconstructive Surgery 120, no. 6 Suppl (2007): 89S–97S. 18090347 10.1097/01.prs.0000248866.57638.40

[41] M. O. Christen , “Collagen Stimulators in Body Applications: A Review Focused on Poly‐L‐Lactic Acid (PLLA),” Clinical, Cosmetic and Investigational Dermatology 15 (2022): 997–1019. 35761856 10.2147/CCID.S359813 PMC9233565

[42] R. Signori , A. P. Barbosa , F. Cezar‐dos‐Santos , et al., “Efficacy and Safety of Poly‐l‐Lactic Acid in Facial Aesthetics: A Systematic Review,” Polymers (Basel) 16, no. 18 (2024): 2564. 39339028 10.3390/polym16182564 PMC11435306

[43] P. F. Jacovella , “Use of Calcium Hydroxylapatite (Radiesse) for Facial Augmentation,” Clinical Interventions in Aging 3, no. 1 (2008): 161–174. 18488886 10.2147/cia.s2065 PMC2544361

[44] S. B. Aguilera , A. McCarthy , S. Khalifian , Z. P. Lorenc , K. Goldie , and W. G. Chernoff , “The Role of Calcium Hydroxylapatite (Radiesse) as a Regenerative Aesthetic Treatment: A Narrative Review,” Aesthetic Surgery Journal 43, no. 10 (2023): 1063–1090. 37635437 10.1093/asj/sjad173 PMC11025388

[45] W. Lee , S. Shah‐Desai , N. K. Rho , and J. Cho , “Etiology of Delayed Inflammatory Reaction Induced by Hyaluronic Acid Filler,” Archives of Plastic Surgery 51, no. 1 (2024): 20–26. 38425859 10.1055/a-2184-6554 PMC10901605

[46] W. Baranska‐Rybak , J. V. Lajo‐Plaza , L. Walker , and N. Alizadeh , “Late‐Onset Reactions After Hyaluronic Acid Dermal Fillers: A Consensus Recommendation on Etiology,” Prevention and Management. Dermatol Ther (Heidelb) 14, no. 7 (2024): 1767–1785. 38907876 10.1007/s13555-024-01202-3 PMC11265052

[47] C. R. Costa , R. Kordestani , K. H. Small , and R. J. Rohrich , “Advances and Refinement in Hyaluronic Acid Facial Fillers,” Plastic and Reconstructive Surgery 138, no. 2 (2016): 233e–236e. 10.1097/PRS.0000000000002008 27465184

[48] B. Liu , Z. Xu , R. Yu , J. Wang , Z. Wang , and C. R. Harrell , “The Use of Type i and Type Iii Injectable Human Collagen for Dermal Fill: 10 Years of Clinical Experience in China,” Seminars in Plastic Surgery 19, no. 3 (2005): 241–250.

[49] L. Salvatore , M. L. Natali , C. Brunetti , A. Sannino , and N. Gallo , “An Update on the Clinical Efficacy and Safety of Collagen Injectables for Aesthetic and Regenerative Medicine Applications,” Polymers (Basel) 15, no. 4 (2023): 1020. 36850304 10.3390/polym15041020 PMC9963981

[50] R. Winters and T. Moulthrop , “Is Autologous Fat Grafting Superior to Other Fillers for Facial Rejuvenation?,” Laryngoscope 123, no. 5 (2013): 1068–1069. 23619619 10.1002/lary.23614

[51] J. A. Kadouch , “Calcium Hydroxylapatite: A Review on Safety and Complications,” Journal of Cosmetic Dermatology 16, no. 2 (2017): 152–161. 28247924 10.1111/jocd.12326

[52] T. Pavicic , “Complete Biodegradable Nature of Calcium Hydroxylapatite After Injection for Malar Enhancement: An MRI Study,” Clinical, Cosmetic and Investigational Dermatology 8 (2015): 19–25. 25709485 10.2147/CCID.S72878 PMC4330000

[53] G. W. Hong , J. Wan , Y. Park , et al., “Manufacturing Process of Hyaluronic Acid Dermal Fillers,” Polymers (Basel) 16, no. 19 (2024): 2739. 39408450 10.3390/polym16192739 PMC11479139

[54] J. Carruthers , S. R. Cohen , J. H. Joseph , R. S. Narins , and M. Rubin , “The Science and Art of Dermal Fillers for Soft‐Tissue Augmentation,” Journal of Drugs in Dermatology 8, no. 4 (2009): 335–350. 19363852

[55] G. W. Hong , H. Hu , K. Chang , et al., “Review of the Adverse Effects Associated With Dermal Filler Treatments: Part I Nodules, Granuloma, and Migration,” Diagnostics (Basel) 14, no. 15 (2024): 1640. 39125515 10.3390/diagnostics14151640 PMC11311355

[56] A. Braz , L. Colucci , L. Macedo de Oliveira , et al., “A Retrospective Analysis of Safety in Participants Treated With a Hybrid Hyaluronic Acid and Calcium Hydroxyapatite Filler,” Plastic and Reconstructive Surgery. Global Open 12, no. 2 (2024): e5622. 38348461 10.1097/GOX.0000000000005622 PMC10860969

[57] F. Xie , J. Qin , J. Sun , Q. Li , and Y. Xie , “Comparative Evaluation of the Biodegradability and Biocompatibility of Agarose Gel and Hyaluronic Acid Filler,” Aesthetic Plastic Surgery 49, no. 22 (2025): 6324–6332. 40999239 10.1007/s00266-025-05239-3

[58] Y. Xie , S. Wu , S. Li , Q. Li , and H. Zhao , “Evaluation of the Long‐Term Safety and Biodegradability of Hyaluronic Acid Dermal Fillers (YVOIRE (R)) for the Correction of Nasolabial Folds: Two Multicenter, Prospective, Observational Cohort Studies,” Journal of Cosmetic Dermatology 21, no. 6 (2022): 2387–2397. 35357748 10.1111/jocd.14952

[59] M. Y. Hyun , Y. Lee , Y. A. No , et al., “Efficacy and Safety of Injection With Poly‐L‐Lactic Acid Compared With Hyaluronic Acid for Correction of Nasolabial Fold: A Randomized, Evaluator‐Blinded, Comparative Study,” Clinical and Experimental Dermatology 40, no. 2 (2015): 129–135. 25319932 10.1111/ced.12499

[60] Administration, N.M.P . (2025), https://www.nmpa.gov.cn/datasearch/search‐info.html?nmpa=aWQ9Njk5NWM3YTRjMTU5NGUxNmE4Yzk1OTczY2RjMWMzMTYmaXRlbUlkPWZmODA4MDgxODMwYjEwMzUwMTgzOGQ0ODcxYjUzNTQz.

[61] N. J. Mount Royal , Global Survey (International Society of Aesthetic Plastic Surgery (ISAPS), ISAPS International Survey on Aesthetic/Cosmetic Procedures, 2024), 2025. Performed in 2024 [Report], https://www.isaps.org.

[62] Y. Kai , Research on Marketing Strategy of Allergan Aesthetics in China's Medical Aesthetics Market (Shanghai University of Finance and Economics, 2023).

[63] J. Long , X. Li , Y. Gao , et al., “Re‐Engineered Beta‐Agarase for Efficient Agarose Oligosaccharide Preparation With Enhanced Thermostability,” Journal of Agricultural and Food Chemistry 73, no. 21 (2025): 12834–12844. 40378394 10.1021/acs.jafc.4c12797

[64] L. Cherwoo , R. Dhaneshwar , P. Kaur , R. Bhatia , and H. Setia , “Optimizing Agarase Production From Microbulbifer sp. Using Response Surface Methodology and Machine Learning Models,” Environmental Technology 46, no. 20 (2025): 4087–4098. 40186854 10.1080/09593330.2025.2485358

[65] Y. Lee , E. Jo , Y. J. Lee , et al., “A Novel Agarase, Gaa16B, Isolated From the Marine Bacterium Gilvimarinus Agarilyticus JEA5, and the Moisturizing Effect of Its Partial Hydrolysis Products,” Marine Drugs 20, no. 1 (2021): 2. 35049857 10.3390/md20010002 PMC8778308

[66] A. Maharjan and B. S. Kim , “Elucidation of Biochemical Attributes and Enzymatic Activity of Agarase From *Saccharophagus degradans* 2‐40,” Enzyme and Microbial Technology 191 (2025): 110733. 40803215 10.1016/j.enzmictec.2025.110733

[67] D. C. D. Roux , I. Jeacomine , G. Maîtrejean , F. Caton , and M. Rinaudo , “Characterization of Agarose Gels in Solvent and Non‐Solvent Media,” Polymers (Basel) 15, no. 9 (2023): 2162. 37177308 10.3390/polym15092162 PMC10181322

[68] C. Krommelbein , M. Mütze , R. Konieczny , et al., “Impact of High‐Energy Electron Irradiation on Mechanical, Structural and Chemical Properties of Agarose Hydrogels,” Carbohydrate Polymers 263 (2021): 117970. 33858571 10.1016/j.carbpol.2021.117970

[69] D. I. Dumitrascu and A. V. Georgescu , “The Management of Biofilm Formation After Hyaluronic Acid Gel Filler Injections: A Review,” Clujul Med 86, no. 3 (2013): 192–195. 26527945 PMC4462513

[70] A. Sadashivaiah and V. Mysore , “Biofilms: Their Role in Dermal Fillers,” Journal of Cutaneous and Aesthetic Surgery 3, no. 1 (2010): 20–22. 20606988 10.4103/0974-2077.63257 PMC2890130

[71] Y.‐L. Zhang , Z. S. Sun , W. J. Hong , Y. Chen , Y. F. Zhou , and S. K. Luo , “Biofilm Formation Is a Risk Factor for Late and Delayed Complications of Filler Injection,” Frontiers in Microbiology 14 (2024): 1297948. 38260874 10.3389/fmicb.2023.1297948 PMC10800873

[72] F. Urdiales‐Gálvez , N. E. Delgado , V. Figueiredo , et al., “Treatment of Soft Tissue Filler Complications: Expert Consensus Recommendations,” Aesthetic Plastic Surgery 42, no. 2 (2018): 498–510. 29305643 10.1007/s00266-017-1063-0 PMC5840246

[73] M. Strathmann , T. Griebe , and H. C. Flemming , “Artificial Biofilm Model ‐ a Useful Tool for Biofilm Research,” Applied Microbiology and Biotechnology 54, no. 2 (2000): 231–237. 10968638 10.1007/s002530000370

[74] J. Allen and K. Dodou , “Current Knowledge and Regulatory Framework on the Use of Hyaluronic Acid for Aesthetic Injectable Skin Rejuvenation Treatments,” Cosmetics 11, no. 2 (2024): 54.

